# Osteopoikilosis and multiple exostoses caused by novel mutations in *LEMD3 *and *EXT1 *genes respectively - coincidence within one family

**DOI:** 10.1186/1471-2350-11-110

**Published:** 2010-07-09

**Authors:** Sevjidmaa Baasanjav, Aleksander Jamsheer, Mateusz Kolanczyk, Denise Horn, Tomasz Latos, Katrin Hoffmann, Anna Latos-Bielenska, Stefan Mundlos

**Affiliations:** 1 Institute of Medical Genetics, Charité Berlin, Humboldt University, Augustenburger Platz 1, 13353 Berlin, Germany; 2 Center for Medical Genetics in Poznań, ul. Grudzieniec 4, 60-601 Poznań, Poland; 3 Chair and Department of Medical Genetics, University of Medical Sciences in Poznań, ul. Grunwaldzka 55 paw.15, 60-352 Poznań, Poland; 4 Max Planck Institute for Molecular Genetics, Development and Disease, Ihnestraße 63-73, 14195 Berlin, Germany; 5 Department of Radiology and Diagnostic Imaging, Nicolaus Copernicus University, Collegium Medicum, Bydgoszcz, ul. Curie-Skłodowskiej 9, 85-094 Bydgoszcz, Poland; 6 Division of Nephrology, Department of Internal Medicine, University Clinic Leipzig, Philipp-Rosenthal-Str. 27, 04103 Leipzig, Germany

## Abstract

**Background:**

Osteopoikilosis is a rare autosomal dominant genetic disorder, characterised by the occurrence of the hyperostotic spots preferentially localized in the epiphyses and metaphyses of the long bones, and in the carpal and tarsal bones [1]. Heterozygous *LEMD3 *gene mutations were shown to be the primary cause of the disease [2]. Association of the primarily asymptomatic osteopokilosis with connective tissue nevi of the skin is categorized as Buschke-Ollendorff syndrome (BOS) [3]. Additionally, osteopoikilosis can coincide with melorheostosis (MRO), a more severe bone disease characterised by the ectopic bone formation on the periosteal and endosteal surface of the long bones [4-6]. However, not all MRO affected individuals carry germ-line *LEMD3 *mutations [7]. Thus, the genetic cause of MRO remains unknown. Here we describe a familial case of osteopoikilosis in which a novel heterozygous *LEMD3 *mutation coincides with a novel mutation in *EXT1*, a gene involved in aetiology of multiple exostosis syndrome. The patients affected with both *LEMD3 *and *EXT1 *gene mutations displayed typical features of the osteopoikilosis. There were no additional skeletal manifestations detected however, various non-skeletal pathologies coincided in this group.

**Methods:**

We investigated *LEMD3 *and *EXT1 *in the three-generation family from Poland, with 5 patients affected with osteopoikilosis and one child affected with multiple exostoses.

**Results:**

We found a novel c.2203C > T (p.R735X) mutation in exon 9 of *LEMD3*, resulting in a premature stop codon at amino acid position 735. The mutation co-segregates with the osteopoikilosis phenotype and was not found in 200 ethnically matched controls. Another new substitution G > A was found in *EXT1 *gene at position 1732 (cDNA) in Exon 9 (p.A578T) in three out of five osteopoikilosis affected family members. Evolutionary conservation of the affected amino acid suggested possible functional relevance, however no additional skeletal manifestations were observed other then those specific for osteopoikilosis. Finally in one member of the family we found a splice site mutation in the *EXT1 *gene intron 5 (IVS5-2 A > G) resulting in the deletion of 9 bp of cDNA encoding three evolutionarily conserved amino acid residues. This child patient suffered from a severe form of exostoses, thus a causal relationship can be postulated.

**Conclusions:**

We identified a new mutation in *LEMD3 *gene, accounting for the familial case of osteopoikilosis. In the same family we identified two novel *EXT1 *gene mutations. One of them A598T co-incided with the *LEMD3 *mutation. Co-incidence of *LEMD3 *and *EXT1 *gene mutations was not associated with a more severe skeletal phenotype in those patients.

## Background

Osteopoikilosis is a rare and primarily benign autosomal dominant genetic entity caused by heterozygous mutations in the *LEMD3 *gene. It is characterised by the occurrence of the hyperostotic spots throughout the skeleton, with most frequent localization in the epiphyses and metaphyses of the long bones, as well as in the carpal and tarsal bones [1]. The clinical features of osteopoikilosis are relatively mild, therefore the condition is usually diagnosed by chance or because of the association with other medical problems (fractures, joint dislocations, etc.). In addition to spotty bone changes, some patients affected by osteopoikilosis develop the superficial skin lesions (elastic-type nevi) and/or subcutaneous foci of dermatofibrosis. Such combination of clinical features is categorized as a separate condition named the Buschke-Ollendorff syndrome [3]. Osteopoikilosis has also been found in association with a more severe and detrimental bone disease called melorheostosis. Melorheostosis manifests with predominantly asymmetric depositions of dense compact bone on the periosteal and endosteal surface of the long bones, resembling a dripping wax of a candle. Bone deformations are often associated with the ossification of the soft tissue in the joint proximity, which can cause compression of the adjacent nerves, and result in pain. Heterozygous *LEMD3 *gene mutations were detected in all such cases. In contrast, no germline *LEMD3 *mutations were found in the isolated cases of melorheostosis [7,8]. Thus, genetic cause of isolated melorheostosis remains unknown. Melorheostosis belongs to a group of osteogenic lesions together with another disease called hereditary multiple exostoses (HME) [9]. Multiple exostoses (enchondromas) are caused by heterozygous mutations in *EXT1*, *EXT2 *and/or *EXT3 *genes. The EXT proteins function in the proteoglycan synthesis and play tumour suppressor roles. *EXT1 *and *EXT2 *have both been shown to encode a heparan sulphate polymerase with both D-glucuronyl (GlcA) and N-acetyl-D-glycosaminoglycan (GlcNAC) transferase activities and their functions are indispensable for heparin-sulphate biosynthesis [10]. The nature of the tumour suppressor effects of the heparan sulphate biosynthesis is not entirely clear, however the regulation of Ihh signalling was proposed to play an important role [11]. Here we describe a family with 5 patients affected by osteopoikilosis caused by novel mutation in the *LEMD3 *gene. Interestingly, three of the patients affected with this new *LEMD3 *mutation additionally carry a new mutation in the *EXT1 *gene. We discuss possible implications.

## Methods

### Patients

We studied a two-branch family of Polish descent. The main branch comprised three generations with five individuals affected by osteopoikilosis. We also examined a more distant kindred affected by the severe deformational condition of the long bones. The local ethics committee approved the study and written, informed consent was obtained from all participants or their legal guardians for publication of this case report, including clinical data, pedigree and X-ray images. Copies of all written consents are available for review on request.

### DNA sequencing

The *LEMD3 *and *EXT1 *genes were analyzed by bidirectional sequencing with primers listed in the see (Table 1). PCR amplification of *LEMD3 *gene was performed in a 20 μl final volume, contained 1 U of Taq polymerase (FIRE Pol) with buffer supplemented with 1.5 mM Mg^2+^, 0.4 mM dNTP, 8 pmol of each forward and reverse primer, and 30 ng of DNA. The exons were amplified as following: 5 min at 94°C, 35 cycles (30 sec 94°C, 30 sec 60°C, 30 sec 72°C) 10 min 72°C. PCR amplifications of *EXT1 *gene were performed in a 20 μl final volume, contained 1 U of Taq polymerase (Invitek) with buffer supplemented with 2 mM Mg^2+^, 0.4 mM dNTP, 8 pmol of each forward and reverse primer, and 30 ng of DNA. PCR conditions used were as in case of *LEMD3*, with exception of exon1-2 and exon 6 amplification, where annealing temperature varied from 55°C to 61°C (touchdown PCR). PCR products were sequenced with the DNA Sequencing Kit BigDye™ Terminator v3.0 Cycle Sequencing (Applied Biosystems) on an ABI 3730 automated sequencer. All exons were compared to genomic sequence (NM_014319.3 for *LEMD3 *gene and NM_000127.2 for *EXT1 *gene) and variations were numbered according to Ensembl ENSG00000174106 for *LEMD3 *and ENSG00000182197 for *EXT1*. Healthy control subjects were screened for the identified mutations in the *LEMD3 *and *EXT1 *genes via sequence analysis. cDNA was synthesised using random hexamer primers as described below. PCR amplification of the *EXT1 *cDNA fragments was done with primer pairs listed in (Table 2). The amplification conditions were like for the genomic DNA sequencing. PCR products were sequenced with the DNA Sequencing Kit BigDye™ Terminator v3.0 Cycle Sequencing (Applied Biosystems) on an ABI 3730 automated sequencer.

**Table 1:** Sequences of the primers used for *LEMD3 *and *EXT1 *gene amplification and sequencing.

Exon name	F Primer sequence 5'- 3'	R Primer sequence 5' - 3'
LEMD3_e1-1	CTCAGGTGAGCTCCTCCC	CGACTCGTCCGAGCTGAAG
LEMD3_e1-2	GCGACCTCTCCTACTTACGG	GTCGTCGTCGTCCTCTTCC
LEMD3_e1-3	AGGAGAGGGACCCGGAG	GGGGAGTCCACACTGAAGG
LEMD3_e1-4	AGGAGGGTGTGATCAAGTGG	GCGCAAATAGTCTTTCAGGG
LEMD3_e2	TTTAGCAAAGTACATGCTGGC	TTATACGACAGTTAGGGAATACTCAG
LEMD3_e3	TTCAGATTATGTGGCTTCTGTG	TTCACAAATATAACACTGGACTTGG
LEMD3_e4	TGTGGTTAATGTAATGGTAGTTGTTTG	GGAACAAGAGCGAAACTGTG
LEMD3_e5-6	TTGGAGTAGTGGGAAAATGC	GCTGTGACTTATGTGGCAACC
LEMD3_e7-8	GAAGGTTCATTCCGTTGTGG	AGTTGAGAAGGGTCACAGCTC
LEMD3_e9	CATCTAAATCTTCTTTGAACAAACTCC	CAGAACGAGAGAGTTTTGCC
LEMD3_e10	CTAACCAGGGGTCTGGCTC	TTTGCTTGGAATTTAATGAAAGAG
LEMD3_e11-12	TCTACCTCCTGTTAGTCAACAAGC	TGGTAAAAGACATATGAGCACAAAAC
LEMD3_e13	ATTGCATGGCTCTTGGTTTG	GCTGCCTCACTGCTAAATCC
EXT1_e1-1	TCTTTACAGGCGGGAAGATG	TGTTCCACAAGTGGAGACTCTG
EXT_e1-2	CCAGGTTCTACACCTCGGAC	CTCAGTTCCAGGCTCAAAGG
EXT1_e2	CTGGTGGCTTTCCCGAG	AAGGGAAACCACACCTTCTC
EXT1_e3	AAGCTTCCTTTCCTTCTGGC	CCATGACACAGGTAATTTTCTCC
EXT1_e4	TGCTAGAAGCCAAATGCTATG	TGGACCAATCACACATCCC
EXT1_e5	CTCTGACTGCCACCATCTTTC	AAGCAATCTTCAATGCAGGG
EXT1_e6	ATTTGCTCCAGCATGAGGC	TGAATGAAAGGGAGTAGCAGG
EXT1_e7	GCTGAGATTTCCAGCTCCTC	AACAGGGAGAAGATATCTAGGGC
EXT1_e8	AGATTCCTTCGGTGTTGAGG	CAAGGCACGGCTAAAAGAAG
EXT1_e9	CCGGATTTTGCATTATGAATTAG	ATCAGCAAAACTTAAGCGGG
EXT1_e10	GGGATTCAAAGAATGGGTATG	CTGGGTGGAACAGCTAGAGG
EXT1_e11	TGCTCATTTGCCTGACTCC	ACAATCTGGCTCTGCTGATG

**Table 2:** Sequences of the primers used for *EXT1 *cDNA amplification and sequencing.

PCR name	F Primer sequence 5'- 3'	R Primer sequence 5' - 3'
*EXT1 *cDNA1	GCTGCTCGCCCGCCCTGGGTG	GTGGTGCAAGCCATTCCTAC
*EXT1 *cDNA2	CTCAGCTGGCTCTTGTCTCG	CTCGGTGTAGTCAGGCCAAG
*EXT1 *cDNA3	CTTGTGGAACAATGGTAGG	CCTATGACGGCAGCTTGGTTC
*EXT1 *cDNA4	GTATGATTATCGGGAAATG	CTGGGCACAGTACTGGGACTTGG
*EXT1 *cDNA5	CTGGTCTCTCAGTCCCAGC	GTCCCATCATTGTCTCCTTATAC
*EXT1 *cDNA6	GCCTCCAATCAAAGTGACCC	CTCTGCTGATGAGTGGATCTGC

### Isolation of the primary osteoblast cells

Surgically removed exostosis was placed in alphaMEM medium supplemented with 10% FCS, Penicilin-Streptomycin and Glutamine and transported to laboratory within 24 h. Bone was cleaned of any remaining connective tissue and 3 rounds of collagenase IV (2 mg/ml) digest 1× 5 min. and 2× 20 min. each at 37°C were performed. Cells from the first digestion were discarded whereas second and third digests were pooled and seeded in the alpha-MEM medium. Cells were cultured till confluent.

### RNA isolation and cDNA synthesis

Total RNA was isolated with peqGOLD *TriFast*™ reagent (PeqLab) according to supplied protocol. cDNAs were synthesised from 1 μg total RNAs with SuperscriptII (Invitrogen) according to manufacturer's guidelines.

## Results

### Clinical History

**Figure 1:**
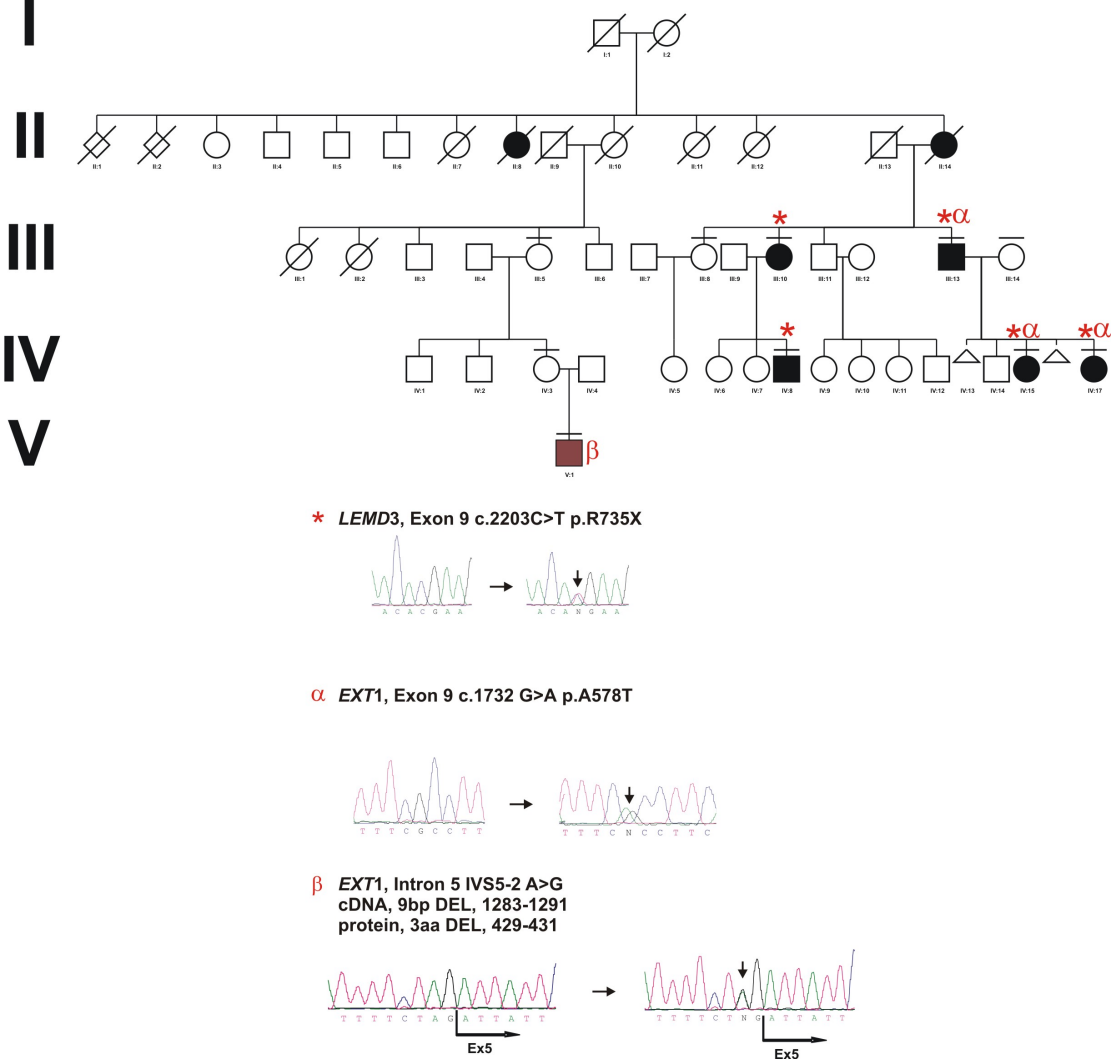
**Co-occurrence of *LEMD3 *and *EXT1 *mutations**. **(A) **Co-occurrence of *LEMD3 *(*) and *EXT1 *missense mutation (α) in the family affected with osteopoikilosis. A 9 bp deletion in *EXT1 *gene (β) affecting patient suffering from multiple exostoses. **(B) **Representative electrophoregrams of the detected mutations. Osteopoikilosis phenotype is shown in black, whereas multiple exostoses syndrome is represented with brown colour. Horizontal bars over symbols mark patients who underwent clinical examination and molecular testing.

**Figure 2:**
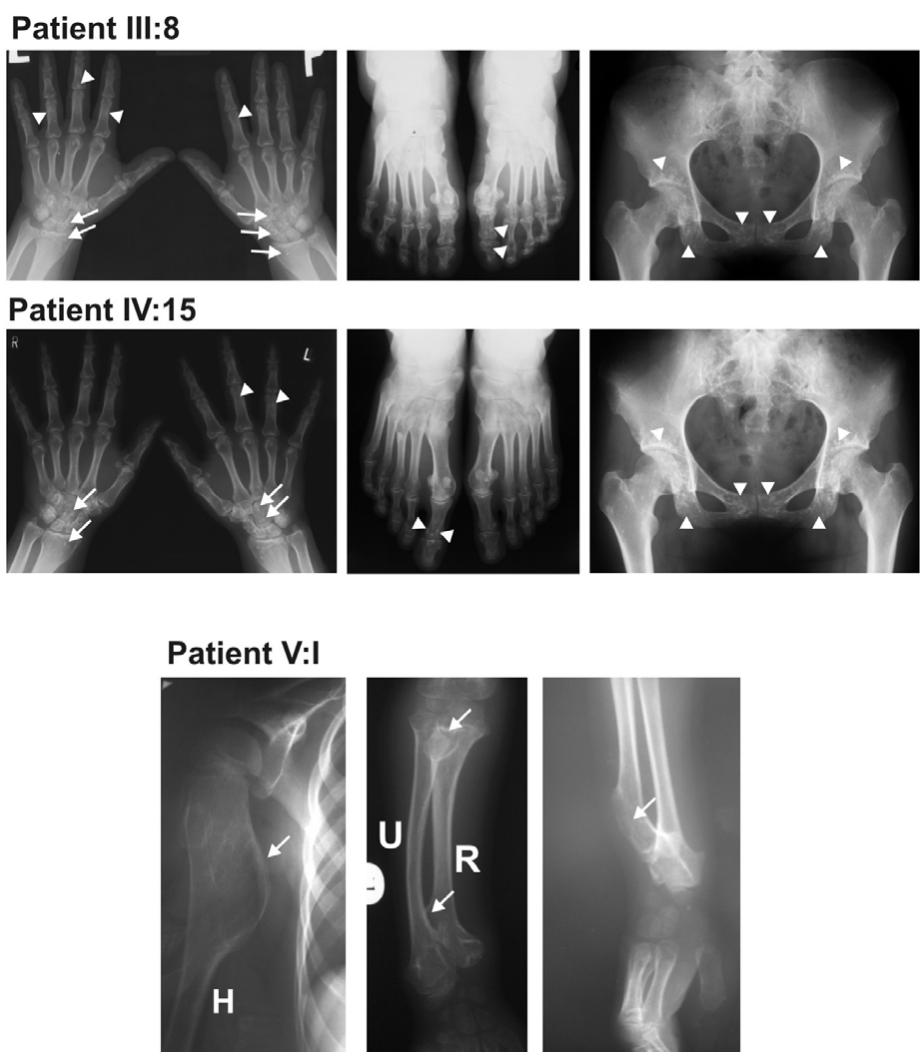
**Sclerotic changes in the hands, feet, and pelvis of the osteopoikilosis affected patients positive for c.2203C > T (p.R735X) *LEMD3 *mutation**. **(A) **Hyperostotic spots are seen bilaterally in the distal parts of radius and carpal bones (arrows) as well as in the phalanges of hands and feet and in the pelvis (arrowheads). **(B) **Radiological appearance of the right hand of the proband (V:I - carrying intron 5 IVS5-2 A > G mutation) at the age of 5 years. Large exostoses in the proximal part of the humerus, as well as in the proximal and distal ends of the ulna and radius are demarcated with arrows.

In the current study we identified a family with congenital osteopoikilosis (Figure 1). The affected family members suffered from moderate to intermittent pain in the hands and feet, with onset of the symptoms varying from 15 (Patient IV:17) to 26 years of age (Patient III:13). X-ray examination revealed disseminated sclerotic foci in the bones of the hands and feet, in the epiphyseal parts of the long bones as well as pelvis and sacrum (Figure 2). Clinical features observed in the affected family members are summarized in (Table 3), and involve several findings: dermatofibrosis, tetralogy of fallot (TOF), ovarian and sinus cysts, diabetes mellitus type 2, and vitiligo. Interestingly these various features were presented by the patients who carried both *LEMD3 *and *EXT1 *gene mutations (with exception of dermatofibrosis which was presented by a patient affected with *LEMD3 *mutation only).

**Table 3:** Clinical symptoms identified in the patients presenting with osteopoikilosis.

	Patient IV:15	Patient IV:17	Patient III:13	Patient III:10	Patient IV:8
	(female; 26 years)	(female; 24 years)	(male; 60 years)	(female; 54 years)	(male; 19 years)
*EXT1 *mutation status	p.A578T	p.A578T	p.A578T	-	-
Painful hands and feet	+	+	+	+	+
Dermatofibrosis	-	-	-	+	-
Additional skin changes	-	-	Vitiligo	-	-
Other symptoms/disorders	TOF, Ovarian cyst	Sinus cyst	DM2	-	-
Laboratory tests (Ca, P, AP, ACP)	NE	NE	NE	Normal	NE

TOF - tetralogy of Fallot; DM2 - diabetes mellitus type 2; NE - not examined; Ca - calcium; P - phosphate; AP - alkaline phosphatase; ACP - acid phosphatase

We also consulted a more distant relative of this family (V:1), who was independently referred to a clinical geneticist at the age of 7 years. X-ray examination showed large multiple exostoses predominantly localized in the ends of the long bones (Figure 2). The boy was operated at the age of 7 years for a large exostosis affecting proximal part of the right humerus. Histopathological examination of the removed bone was suggestive of enchondromatosis.

### Mutation Detection

**Figure 3:**
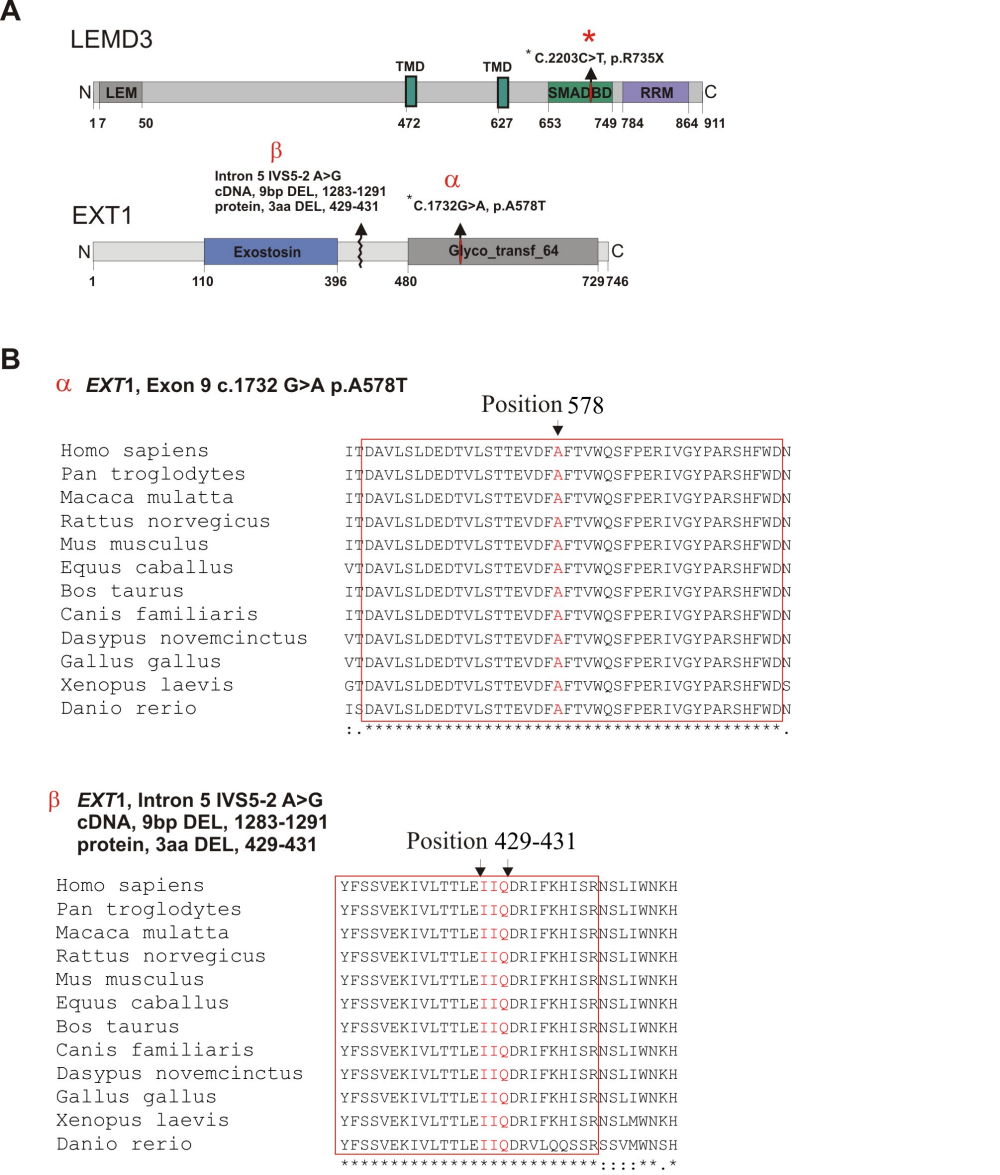
**Schematic representation of the LEMD3 and EXT1 protein structure with the protein motifs and domains assigned**. **(A) **The LEMD3 R735X (*) mutation localizes in SMAD binding domain of LEMD3. EXT1 mutation A578T (α) localizes in the glycotransferase domain of EXT1. EXT1 mutation IVS5-2 A > G (p. DEL 429-431) (β) is located in the conserved region of the protein in-between two catalytically active domains. **(B) **Evolutionary conservation of the mutated amino acid residues in the EXT1. The block of absolute sequence conservation surrounding mutation sites is demarcated with the red bracket.

Full coding sequence and exon-intron boundaries of the *LEMD3 *gene were analyzed in the nine individuals. Patients (III:10, III:13, IV:8, IV:15, IV:17) were heterozygous for C-to-T transition in exon 9 (c.2203C > T) on genomic DNA. This mutation was predicted to change amino acid 735 from an arginine to a stop codon (p.R735X) (Figure 1, Figure 3A). Three out of these patients (III:13, IV:15, IV:17) additionally carried a heterozygous mutation in the *EXT1 *gene. This mutation altered G-to-A in exon 9 (c.1732G > A) on genomic DNA and predicted to change amino acid 578 from an alanine to a threonine (p.A578T). Presence of both *LEMD3 *and *EXT1 *variants was excluded among 81 and 247 healthy Polish and German controls respectively. Bioinformatic analysis of the EXT1 sequence with SIFT http://sift.jcvi.org and PolyPhen http://genetics.bwh.harvard.edu/pph/ software indicated a high probability of the mutation being deleterious for the protein function (PolyPhen - PSIC score difference for A578T: 1.688) - see (Figure 3B) for sequence conservation. However, affected patients did not exhibit exostoses and no additional skeletal manifestations beyond hyper-mineralized foci were detected. Interestingly, patients affected with both *LEMD3 *and *EXT1 *mutations presented spectrum of additional non-skeletal pathologies, which included: (IV:15) TOF and ovarian cysts, (IV:17) sinus cysts, and (III:13) diabetes melitus type 2 and vitiligo (Table 3).

A different mutation in *EXT1 *gene was identified in the patient (V:1), diagnosed with multiple exostoses syndrome. The patient carried a heterozygotic splice site mutation in intron 5 (IVS5-2 A > G), as detected in blood lymphocytes and primary osteoblast progenitor cells obtained from the surgically removed exostoses. Sequencing of the exostoses derived cDNA (obtained from the cells isolated from the affected bone) showed that splice site mutation resulted in the in-frame deletion of 9 bp of the exon 5 leading to a deletion of three amino acids (pos. 429-431 - two conserved isoleucin residues and a conserved glutamic acid residue) (Figure 3B). Functional relevance of the deleted amino acids was predicted based on their evolutionary conservation.

## Discussion

We identified a family with five members affected by osteopoikilosis caused by a novel nonsense heterozygous mutation (p.R735X) localised in exon 9 of the *LEMD3 *gene. In other branch of this family we identified a boy affected by more severe bone deformations. The boy was initially suspected of the melorheostosis, but upon X-ray examination, the diagnosis was corrected to the multiple exostoses syndrome. Clinical diagnosis was subsequently confirmed by sequence analysis of the *EXT1 *gene and identification of a previously undescribed splice site mutation (IVS5-2 A>G). This finding led us to sequence *EXT1 *gene in the rest of the family. Surprisingly, three of the osteopoikilosis affected patients additionally to *LEMD3 *mutation carried a yet unreported amino acid variant (p.A578T) in the *EXT1 *gene. Of note was a wide spectrum of the clinical symptoms observed in these family members, which ranged from heart defect, diabetes mellitus, vitiligo to ovarian and sinus cyst formation. None of these pathologies was observed in the examined family members who carried *LEMD3 *mutation only (patients III:10 and IV:8), nor in the family members who were free of mutations in both genes (patients III:5, III:8, III:14, IV:3). However, since we were unable to examine other unaffected family members, the relevance of this observation remains uncertain. The *EXT1 *splice site mutation and other identified mutations must have occurred independently in the two branches of the family.

The exact mechanism by which *LEMD3 *gene mutations lead to the formation of the bone lesions is not clear. *LEMD3 *inactivation in mice was recently shown to result in the mid-gestation lethality [12]. However, heterozygous mice were healthy and no bone lesions reminiscent of osteopoikilosis could be detected, leaving question mark over patho-mechanism of the disease. Co-occurrence of the *LEMD3 *gene mutation with the mutation in another gene has not yet been reported. Presented case constitutes first such report. Following considerations appear relevant based on the review of the available literature. It has been shown that LEM domain containing proteins interact with the barrier-to-autointegration factor (BAF) [13]. BAF is a component of the chromatin remodelling complex, which uses energy from ATP to dismantle DNA-histone complexes [14]. This is on one hand necessary for initiation of transcription, and it has been postulated that LEMD3, through BAF and SMAD interactions might regulate the expression of osteogenic genes [2]. On the other hand it is known that chromatin remodelling is necessary for the efficient DNA repair [15]. LEMD3 closely associates with the intranuclear lamina and mutations in other lamin interacting proteins are known to result in the DNA damage accumulation [16]. Indeed, it has been suggested that lamin complexes acts as assembly scaffolds for DNA repair machinery [17]. Thus, it seems legitimate to ask if inactivation of LEMD3 could also result in an increased mutational susceptibility and increased frequency of the post-zygotic second hit mutation occurrence. In this context it is interesting to note that osteopoikilosis was previously reported to coincide with other pathological entities, including various types of cancers: synovial chondromatosis [18], synoviosarcoma [19], chondrosarcoma [20], osteosarcoma [21], giant cell tumor [22], metastatic breast carcinoma [23], as well as developmental dysplasias: dental, facial abnormalities, coarctation of the aorta, double urether, mental retardation and other reviewed by Gunal et.al. [24].

Clearly, further research is needed to address possible association of the *LEMD3 *loss of function with DNA mutation susceptibility. Presented study constitutes first example of the *LEMD3 *gene mutation co-occurrence with additional genetic alteration, which could potentially modify and/or constitute the nature of the osteopoikilosis.

## Conclusions

The presented case points to importance of the thorough clinical evaluation of the osteopoikilosis patients as phenotypic features of osteopoikilosis with melorheostosis might be confused with the co-occurrence of osteopoikilosis and multiple exostoses. The data encourage re-evaluation of the known osteopoikilosis families for the possible co-occurrence of other than Buschke-Ollendorff and melorheostosis disease entities and investigation of the possible LEMD3 function in the DNA repair.

## Competing interests

The authors declare that they have no competing interests.

## Authors' contributions

SV: performed sequencing and helped in manuscript preparation AJ: Consulted the family, collected and processed clinical material, conceived the manuscript. MK: coordinated sample processing, performed histological analysis of the surgically removed exostosis material, isolated primary cells from the exostoses tissue material and prepared DNA out of primary cells, and conceived the manuscript. DH: provided expert consultations critical in diagnosing multiple exostoses syndrome. KH: provided advice on sequencing, nuclear envelope proteins and helped in manuscript preparation. TL: Referred the family to a clinical geneticist. ALB: consulted the family, critically revised the manuscript. SM: critically revised the manuscript. All authors read and approved the final manuscript.

## Pre-publication history

The pre-publication history for this paper can be accessed here:

http://www.biomedcentral.com/1471-2350/11/110/prepub
